# Valorization of textile waste through biodegradation, fruiting body production, and mycelium composite formation by *Pleurotus ostreatus*

**DOI:** 10.3389/fbioe.2026.1907544

**Published:** 2026-10-08

**Authors:** Norbert Lipka, Kristian Marzec, Karol Bańcer, Arkadiusz Głowacki, Wiesław Kaca

**Affiliations:** 1 Department of Microbiology, Institute of Biology, Jan Kochanowski University, Kielce, Poland; 2 VIVE Innovation, Warsaw, Poland; 3 Faculty of Biotechnology and Gardening, Agriculture University, Krakow, Poland

**Keywords:** composites, Mycelium, Recycling, textile, waste

## Abstract

Waste textiles, particularly those containing natural fibers such as cotton, linen, and hemp, can be repurposed as substrates for oyster mushroom (*Pleurotus ostreatus*) cultivation due to their high cellulose and lignin content. This approach offers an environmentally friendly strategy for textile waste valorisation while simultaneously producing edible mushrooms and mycelium-based composite materials with potential insulating applications. In the present study, cultivation conditions for *P. ostreatus* were optimized with respect to growth-medium composition, humidity, temperature, and fruiting parameters. Both mycelium and fruiting bodies were analysed for metal content. Among the metals tested, only zinc (Zn), iron (Fe), and copper (Cu) were detected in both mycelium and fruiting bodies, at comparable concentrations of approximately 170, 80 and 21 mg/kg, respectively. Across six experimental variants, fruiting body yields ranged from 0 to 45 g per cultivation bag, with an average yield of 20 g per bag. A schematic production workflow for *P. ostreatus* fruiting bodies based on substrates containing 30%, 50%, and 100% textile waste is presented. In addition, selected mechanical and thermal properties of textile–mycelium composites were evaluated, demonstrating their potential applicability as bio-based construction and insulation materials.

## Graphical Abstract

**Figure:**
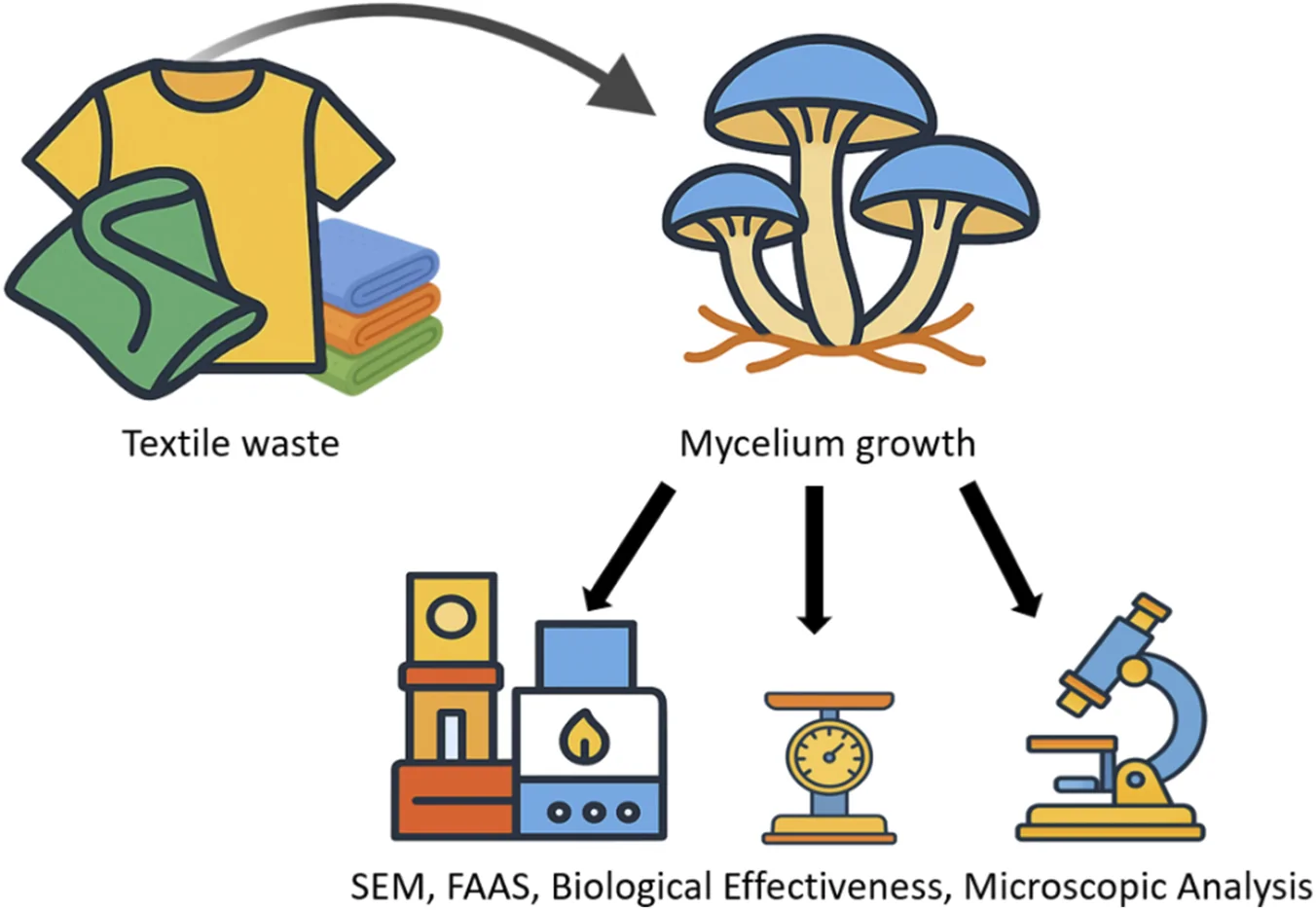

## Highlights

- Waste textiles containing natural fibers can be successfully reused as a substrate for *Pleurotus ostreatus* cultivation.
- Textile-based substrates provide a sustainable pathway for simultaneous mushroom production and textile waste valorization.
- Optimal environmental parameters (substrate composition, humidity, temperature, fruiting conditions) were established for effective mycelial growth and fruiting.
- Only Zn, Fe, and Cu were detected in mycelium and fruiting bodies, with comparable concentrations in the ppm range.
- Mycelium–textile composites exhibited promising mechanical and thermal properties, indicating potential for insulating building materials.

## 1 Introduction

*Pleurotus ostreatus* (oyster mushroom) is the second most widely cultivated edible mushroom worldwide, after *Agaricus bisporus* (button mushroom) (Grimm et al., 2018). Owing to its high nutritional value, adaptability to diverse substrates, and relatively simple cultivation requirements, *P. ostreatus* has attracted considerable scientific and industrial interest. Numerous studies have focused on optimizing cultivation conditions and enhancing fruiting body yield (Kimenju et al., 2009; Llanaj et al., 2023).

Commercial mushroom production is traditionally based on lignocellulosic substrates such as straw, sawdust, and wood chips. Through this process, mushroom-forming fungi convert low-value agricultural waste into high-value food products, while the spent mushroom substrate can be further utilized for composting or other industrial applications (Lim, Lee, and Kang, 2013).

A broad range of substrates has been investigated for *P. ostreatus* cultivation. The mycelium can grow efficiently on various organic wastes, including agricultural residues, food waste (e.g., vegetable peels and spent coffee grounds), and lignocellulosic by-products. Recent studies have also demonstrated that mycelium can grow on textile waste when combined with organic substrates. For example, leather-like fungal materials have been produced following post-processing of *P. ostreatus* mycelium with heat, glycerol, and wax (Sangosanya and Pistofidou, 2024). Other alternative substrates have been explored to produce mycelium-based composites (MBCs). Mixtures of pineapple fibers and spent coffee grounds have been shown to support mycelial growth and composite formation, with a 30% pineapple fiber content resulting in the highest bending stress. Importantly, these MBCs exhibited non-combustible properties, highlighting their potential application as sustainable construction materials (Kohphaisansombat et al., 2024).

Lignocellulosic biomass represents the most abundant renewable resource on Earth and is generated in large quantities as a by-product of agriculture, fruit production, and forestry. Consequently, lignocellulosic substrates are commonly used for *P. ostreatus* cultivation. Numerous studies have examined mycelial growth and fruiting optimization using such materials. Effective cellulose degradation by *P. ostreatus* has been reported on media composed of winery and apple processing waste (1:1 ratio), demonstrating the suitability of these residues for mushroom compost preparation and fruiting body production (Ravlikovsky et al., 2025).

Despite growing interest in fungal biomaterials, research on textile-based biocomposites produced using *P. ostreatus* mycelium remains limited. Previous studies reported maximum compressive strengths of approximately 270 kPa for cotton textile waste and 100 kPa for polyester textile waste. Mycelial growth on textile substrates has been confirmed using scanning electron microscopy (SEM) and Fourier-transform infrared spectroscopy (FTIR), which identified fatty acids, amides, and polysaccharides as key components of the resulting composites. Fungal growth resulted in a 1%–5% decrease in dry mass, which was attributed to mycelium deactivation and drying at 105 °C for 24 h (Saini, Kaur, and Brar, 2024).

The use of fungal mycelium to create biocomposites has primarily been described for plant-based substrates. In these systems, mechanical properties are influenced by fungal species, substrate composition, and growth conditions (Yang et al., 2021; Girometta et al., 2019). A search of patent databases using the keyword combination “textile–mycelium” did not reveal any inventions directly addressing the use of textile waste for mycelium-based materials (Cerimi et al., 2019) (EPO Vive Textile recycling n.d.). While numerous mycelium applications have been described, including building materials and packaging, textile-containing substrates have rarely been reported (Yang et al., 2021 Lim et al., 2013; Girometta et al., 2019; Ali et al., 2023).

The application relates to guanosine tetraphosphate (ppGpp, alarmone) extracted from *Trichoderma viride* mycelium. ppGpp is one of the compounds responsible for environmental stress response in plants. The patent claim describes a complex procedurefor extracting and identifying the alarmone from *T. viride* mycelium grown under various light exposure conditions (Method for Obtaining and Extracting Secondary Fungal Metabolites, Patent, PL 444437 A1). Another patent describes a method for industrial-scale production of *Fomitopsis officinalis* mycelium in liquid cultures. *Fomitopsis officinalis* mycelium has anticancer and anti-inflammatory properties (Patent Application, PL 438480 A1).

Spent mushroom compost (SMC), consisting of residual mycelium remaining after fruiting, has also been investigated as a valuable secondary resource (Dąbrowska et al., 2023). For example, SMC derived from *P. ostreatus* cultivation has been used to isolate lignocellulolytic enzymes such as amylase, cellulase, laccase, and xylanase, which were subsequently applied for the decolorization of Congo Red dye. (Lim et al., 2013).

Macrofungal have been proposed for a wide range of industrial applications, including biodegradable alternatives to plastics, thermal and acoustic insulation materials, components for construction and infrastructure, microbial fuel cells for renewable energy production, and sustainable packaging (Elkhateeb, Elnahas, and Daba, 2021).

One comparatively unexplored application is the recycling of textile waste. In this context, *P. ostreatus* has been cultivated on multiple textile substrates using different growth media, with potato dextrose yeast agar yielding the highest biomass. These studies demonstrated that a variety of textile fabrics can support mycelial growth, although early-stage growth conditions were critical for successful cultivation. Morphological characteristics of the mycelium were strongly influenced by both fabric composition and growth medium (Fletcher, 2025). Other fungi have also shown potential for textile waste bioconversion. The white-rot fungus *Hypholoma fasciculare* has been reported to grow on semi-synthetic textiles while simultaneously bioremediating azo dyes (Harper and Moody, 2023). Macrofungi *Pleurotus ostreatus*, *Pleurotus eryngii*, *Lentinula edodes*, and *Agaricus bisporus* were able to grow on mixed-fibre textile waste composed of cotton, bamboo viscose, and elastane, and to metabolize the textile dye Reactive Black 5 (Hazelgrove and Moody, 2024).

Globally, textile waste represents a major environmental challenge (Suen et al., 2023). More than 85% of textile waste in the United States and approximately 73% in the United Kingdom enters municipal waste streams and is ultimately incinerated or landfilled (Damayanti et al., 2021; Ribul et al., 2021; Harmsen, Scheffer, and Bos, 2021). Globally, only about 1% of textile waste is recycled (Zhao et al., 2021). Textile waste recycling depends on socio-economical statues of consumers (Mim et al., 2025). Decomposition of textiles in landfills depends on the presence of suitable microorganisms. Experimental inoculation of ten textiles types with fungal species by *Chaetomium globosum* (Ascomycota), *Trametes versicolor* and *Ganoderma* species (Basidiomycota) has resulted in complete degradation of cellulose-based materials, whereas in blended textiles containing polyester or elastane, only the cellulose fraction was degraded (Freeman et al., 2024).

The key results the present study indicates that *P. ostreatus* can grow in both polyesters and cotton textiles and form biocomposites with compressive strength between 100 and 270 kPa. It show the ability of the fungus to thrive on polyester plastic in textiles (Burgada et al., 2021; Mysiurkiweicz et al., 2023).

In the present study, *Pleurotus ostreatus* was cultivated on mixed, unsorted textile waste combined with agricultural waste in the form of oak wood pellets. Both mycelial growth and fruiting body production were successfully achieved. Cultivation parameters were optimized with respect to substrate composition, temperature, humidity, lighting, and cultivation time to support both mycelium-based composite formation and mushroom yield. Selected mechanical and thermal properties of the resulting textile–mycelium composites were also investigated to assess their preliminary material characteristics and their potential for further development as bio-based insulation materials.

## 2 Materials and Methods

A schematic representation of the entire experimental workflow is shown in Figure 1. Post-consumer textile waste supplied by VIVE Textile Recycling Sp. z o.o. was used as a component of the cultivation substrate. Approximately 1 kg of used clothing was initially screened for fibre quality and subsequently shredded using a knife mill to obtain fractions smaller than 15 mm. Metallic and other hard elements (e.g., buttons and fasteners) were removed, and the textile material was homogenized in a mixing tank equipped with a vertical ribbon mixer.

**FIGURE 1:**
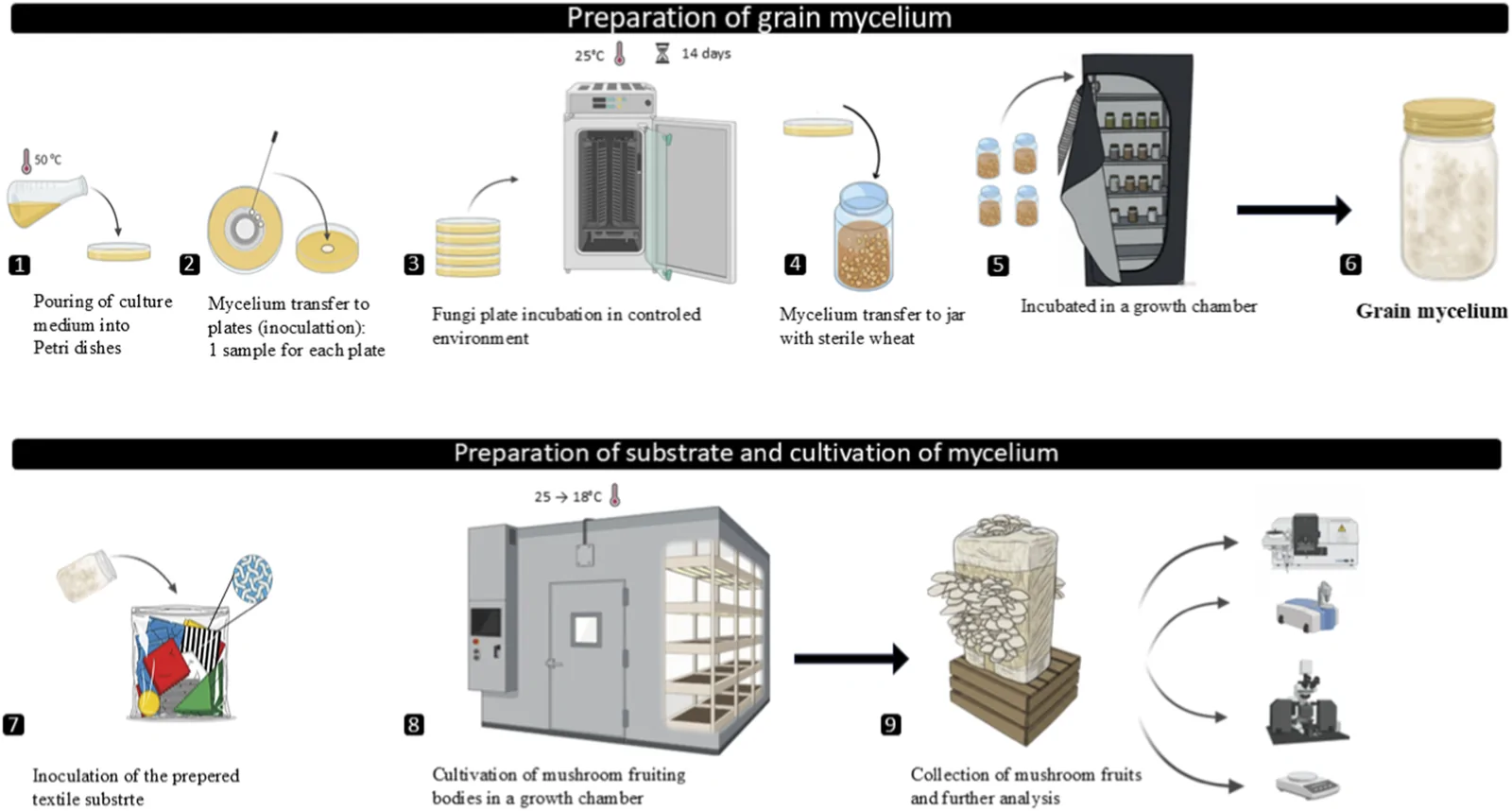
Experiments scheme of *Pleurotus ostreatus* cultivation on textile waste-oak wood pellets substrates.

Four substrate formulations were prepared and inoculated with *Pleurotus ostreatus* grain spawn. Six independent biological culture replicates were prepared for each substrate composition. The biological replicates were treated as independent experimental units for measurements relating to the culture. Mushroom cultivation was carried out according to the procedure described in the Materials and Methods section. Fruiting bodies were harvested from each substrate for two flushes.

Biological efficiency (BE), defined as the ratio of the total fresh weight of harvested fruiting bodies (summed across both flushes) to the dry weight of the substrate excludes spawn mass (expressed as a percentage), was calculated for each experimental variant. The results are summarized in Table 1.

**TABLE 1:** Biological efficiency of oyster mushroom cultivation on a textile–wood substrate. Values are means of six replicates $\left(n=6\right)$ , expressed per 1 kg of dry substrate.

Substrate composition	Mean ( $\bar{x}$ )	Standard Deviation [g]	Standard Error	Margin of Error (95%)	Confidence interval (95%)
First fruiting body harvest [g]					
100% textiles	132.42	4.31	1.76	4.52	[127.90; 136.94]
50% textiles/50% pellets	154.72	5.03	2.05	5.28	[149.44; 160.00]
30% textiles/70% pellets	180.72	5.87	2.4	6.16	[174.56; 186.88]
100% wood pellets	285.69	9.28	3.79	9.74	[275.95; 295.43]
Second fruiting body harvest [g]					
100% textiles	62.91	2.05	0.83	2.15	[60.76; 65.06]
50% textiles/50% pellets	83.68	2.72	1.11	2.85	[80.83; 86.53]
30% textiles/70% pellets	104.28	3.39	1.38	3.56	[100.72; 107.84]
100% wood pellets	266.11	8.65	3.53	9.07	[257.04; 275.18]
Biological yield (%)					
100% textiles	19.53	0.64	0.26	0.67	[18.86; 20.20]
50% textiles/50% pellets	23.84	0.77	0.32	0.81	[23.03; 24.65]
30% textiles/70% pellets	28.5	0.93	0.38	0.97	[27.53; 29.47]
100% wood pellets	55.18	1.79	0.73	1.88	[53.30 57.06]

### 2.1 Fungal strain and culture maintenance

The *Pleurotus ostreatus* strain used in this study was obtained from the fungal culture collection of the University of Agriculture in Kraków. The fungus was maintained on potato dextrose agar (PDA) and incubated at 25 °C until use.

### 2.2 Textile waste

Post-consumer textile waste was supplied by VIVE Textile Recycling sp. z o.o. (Poland, patent PL/EP 2303534). Before shredding, hard non-textile items, including buttons and fastenings, were removed. The material was mechanically shredded to produce particles smaller than 15 mm. No additional washing, dry-cleaning or decontamination was carried out, as the aim was to assess a realistic, mixed stream of post-consumer textile waste rather than a purified fibre fraction.

The fibre composition was estimated based on the fibre composition information provided on the clothing labels. The resulting textile fraction consisted mainly of polyester (approximately 58%), followed by cotton (approximately 25%), nylon (approximately 4.5%) and other fibres. As this composition was determined on the basis of clothing labels rather than gravimetric fibre separation, these values should be treated as approximate estimates rather than direct analytical measurements of the mass fractions of the fibres.

A particle size of less than 15 mm was selected to facilitate homogeneous mixing with the oak wood pellet fraction, whilst maintaining a particle structure suitable for colonisation of the substrate by P. ostreatus. Particle size may also influence the substrate’s porosity, water retention and aeration; it is therefore considered one of the process parameters affecting fungal growth.

### 2.3 Cultivation media

Oak wood pellets were purchased from an open-market supplier (EKO-GONT Piela Damian, Poland).

The wood pellets were characterized by a total moisture content of 6.1%, an ash content of 0.5%, and a sulfur content of 0.016%.

*Pleurotus ostreatus* was cultivated on four substrate formulations with different proportions of oak wood pellets and textile waste:

- Control: 100% oak wood pellets
- 70% oak wood pellets + 30% textile waste
- 50% oak wood pellets + 50% textile waste
- 100% textile waste

All substrate compositions are expressed on a dry-weight basis.

### 2.4 Cultivation of Pleurotus ostreatus on oak wood pellets and textile waste

Wheat grains were soaked in tap water and boiled until approximately 50% hydration was achieved. The hydrated grains were transferred into 500 mL glass flasks and sterilized at 121 °C and 15 psi for 60 min, followed by cooling to room temperature. Sterilized grains were inoculated under aseptic conditions with agar plugs colonized by *P. ostreatus*. The inoculated flasks were incubated in darkness at 25 °C for 7 days and periodically shaken to ensure uniform redistribution of the mycelium throughout the substrate. Incubation continued until complete colonization of the grains, resulting in fully developed grain spawn.

Substrate mixtures were prepared in batches corresponding to 1 kg of dry material. Distilled water was added to achieve a final moisture content of approximately 60%. Each batch was transferred to an autoclavable polypropylene cultivation bag and sterilized in an autoclave at 121 °C for 2 h. After cooling, the substrates were inoculated with grain spawn under a laminar airflow cabinet at a rate of 5% (w/w), relative to the dry weight of the substrate.

Following inoculation, the substrates were thoroughly mixed, and the bags were sealed using a heat-sealing machine. The inoculated substrates were incubated in darkness at 25 °C until full colonization. To induce fruiting body formation, the bags were transferred to a controlled growth chamber and perforated using a sharp, disinfected knife. The temperature was gradually reduced to 18 °C ± 1 °C. A ventilation system supplied fresh air and reduced the CO_2_ concentration to approximately 900 ± 50 ppm. Relative humidity was increased to 90% ± 2%, and illumination was maintained at 500 ± 30 lux. These environmental conditions were maintained throughout fruiting body development until harvest.

### 2.5 Scanning electron microscopy and energy-dispersive X-ray spectroscopy (SEM/EDS)

Qualitative and semi-quantitative elemental analyses were performed using scanning electron microscopy coupled with energy-dispersive X-ray spectroscopy (SEM/EDS). Analyses were conducted on specific components of the final composite, including isolated vegetative mycelium, separated fruiting bodies, and the raw post-consumer textile fibers. A JEOL JSM-6490 LV scanning electron microscope equipped with an EDS microanalyzer was used. Measurements were conducted at an accelerating voltage of 20 kV, and secondary electron imaging (SEI) was applied to obtain micrographs of the sample surfaces. SEM/EDS analyses were carried out as qualitative and semi-quantitative measurements of selected surface areas, rather than as elemental analyses of the bulk material. Representative areas were selected from the examined sample surfaces in order to characterise differences in morphology and elemental composition between the experimental variants. Due to the low electrical conductivity of the organic and synthetic matrices, the surfaces of the mycelium, fruiting body, and textile samples were coated with a thin metallic layer prior to analysis. Gold sputtering was performed using a JEOL JFC-1300 sputter coater, producing an approximately 10 nm thick gold layer. Consequently, the gold-related peaks originating from the conductive coating were excluded from the interpretation of the elemental composition of the original samples.

### 2.6 Flame atomic absorption spectrometry (FAAS)

Quantitative determination of selected metals was performed using flame atomic absorption spectrometry (FAAS). Prior to analysis, distinct samples of harvested fruiting bodies and separated mycelium matrices were isolated and subjected to microwave digestion. Digestion was carried out using a Milestone UltraWAVE microwave digestion system operating in single reaction chamber (SRC) mode. The digestion process was conducted in a sealed pressure chamber under an inert gas atmosphere. During digestion, the vessels containing the biological biomass samples were immersed in liquid, ensuring uniform temperature and pressure conditions across all analytical replicates. For each material tested, two parallel analytical samples were measured, and the value given represents their arithmetic mean. Results below the applicable analytical detection limit are indicated by the symbol ‘<’. As this study was primarily exploratory in nature and aimed to characterise the elemental composition of biological materials, the results obtained using the FAAS method should be interpreted with particular caution in the case of concentrations close to the analytical detection limit. The lack of Certified Reference Material (CRM) was due to the nature of the samples, which consisted of waste from consumer textiles.

### 2.7 Determination of ash content

Ash content (inorganic fraction) was determined by the gravimetric method following dry mineralization. Separate, finely ground specimens of the raw textile substrate, cultivated vegetative mycelium, and mature fruiting bodies were heated in a Nabertherm L15/11/B170 muffle furnace at temperatures not exceeding 600 °C, using platinum crucibles. For each material, two parallel samples with masses of approximately 0.5–1.0 g were analysed.

### 2.8 Biological efficiency

Fruiting bodies were harvested from mycelium cultivated as described above (2.4). The harvested mushrooms were weighed using an Axis ACZ320 precision balance. Between successive fruiting cycles, the substrates were incubated in darkness at 7 °C for 1 week. The bags were then returned to the growth chamber to initiate subsequent fruiting body development.

Biological efficiency (BE) was calculated as the ratio of the cumulative total fresh weight of harvested fruiting bodies grown on material to the dry weight of the substrate, expressed as a percentage:

$$\text{BE }\left(\%\right)=(\text{fresh weight of fruiting bodies }\left[g\right]/\text{ dry weight of substrate }\left[g\right])\times 100$$

### 2.9 Thermal analysis

Thermal analysis of the mycelium was performed using a STA 449 F3 Jupiter thermogravimetric analyzer (TGA, Netzsch, Selb, Germany). Measurements for the mycelium were performed in the temperature range from 25 °C to 650 °C, with a sample weight of 5 ± 1 mg. The samples were placed in an open crucible made of Al_2_O_3_. The analyses were carried out in an atmosphere of oxygen and nitrogen with a flow rate of 20/40 µL min^-1^, respectively, at a constant heating rate of 10 °C min^-1^. Data recording and analysis were performed using *Proteus* Thermal Analysis 8.0.3 software (Netzsch, Selb, Germany). Based on the obtained thermograms, parameters characterizing the thermal stability of the tested materials were determined, including the temperature of 5% mass loss of the sample (T_5_), the temperature of 50% mass loss (T_50_), the temperature of maximum thermal decomposition rate (T_RMAX_), the thermal decomposition rate (dm/dt), the temperature range of combustion of residues after thermal decomposition (ΔT_s_), residues after thermal decomposition (P_TD_), and the content of residues at 600 °C (P_600_).

### 2.10 Determination of mechanical properties in compressive strength testing

Mechanical tests were carried out to determine the preliminary properties of the manufactured textile-mycelium composites. Tensile strength tests were conducted using a Galdabini Quasar 50 testing machine under standard laboratory conditions at a relative humidity of 50 ± 5 per cent. The procedure was based on the ASTM D1621 standard and was modified to accommodate the geometry and heterogeneous structure of the manufactured profiles. Five specimens were tested for each type of composite. As the materials under test exhibited a non-uniform, porous structure, the mechanical properties obtained are treated as comparative data rather than a full, standardised qualification of the material for construction applications.

## 3 Results

### 3.1 Production of P. ostreatus mycelium with textile waste and biological efficiency of oyster mushroom cultivation

All biological experiments were carried out in six independent biological replicates. The values obtained should be interpreted as exploratory results requiring further statistical validation using a larger number of samples.

Table 1 presents the biological efficiency of *P. ostreatus* cultivation on substrates containing different proportions of textile waste and oak wood pellets. A total of 24 substrate units were prepared, corresponding to six replicates of each formulation: (i) control substrate containing 100% oak wood pellets, (ii) 70% oak wood pellets and 30% textile waste, (iii) 50% oak wood pellets and 50% textile waste, and (iv) 100% textile waste.

The highest biological efficiency was observed for the control substrate containing only oak wood pellets (55.18%). Substrates containing textile waste showed progressively lower biological efficiency with increasing textile content. The substrate composed entirely of textile waste exhibited the lowest biological efficiency (19.53%). These results indicate that the presence of oak wood pellets was associated with higher fruiting body production, and that increasing textile waste content led to a reduction in biological efficiency.

### 3.2 SEM/EDS analysis

SEM/EDS analysis was performed to investigate the elemental composition of the mycelium, fruiting bodies, and textile substrates. The analysed samples included:

1. Mycelium samples (M) after cultivation:
o M0%TX (100% oak wood pellets),
o M30%TX (70% oak wood pellets + 30% textile waste),
o M50%TX (50% oak wood pellets + 50% textile waste),
o M100%TX (100% textile waste).
2. Fruiting body samples (F) harvested from substrates containing textile waste:
o F0% (control substrate without textile waste),
o F30% (30% textile waste),
o F50% (50% textile waste),
o F100% (100% textile waste).
3. Textile waste samples (100%TX) prior to cultivation.

Representative SEM images and EDS spectra of the analysed samples are shown in Figures 2–4, including both initial samples and ash samples obtained after mineralization at 600 °C.

**FIGURE 2:**
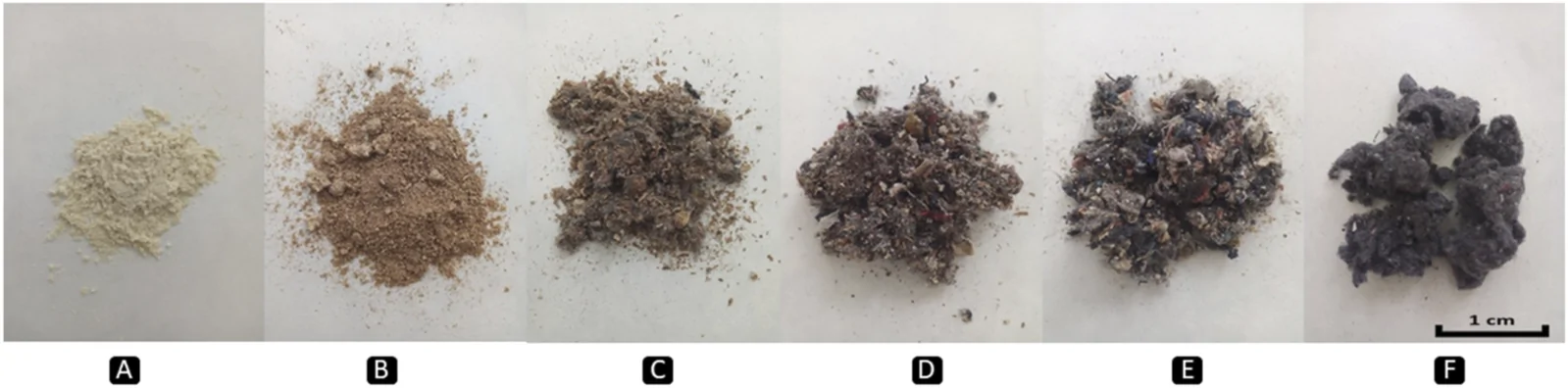
Samples of the tested material **(A)** F30%; **(B)** M0%TX; **(C)** M30%TX; **(D)** M50%TX; **(E)** M100%TX; **(F)** 100%TX.

**FIGURE 3:**
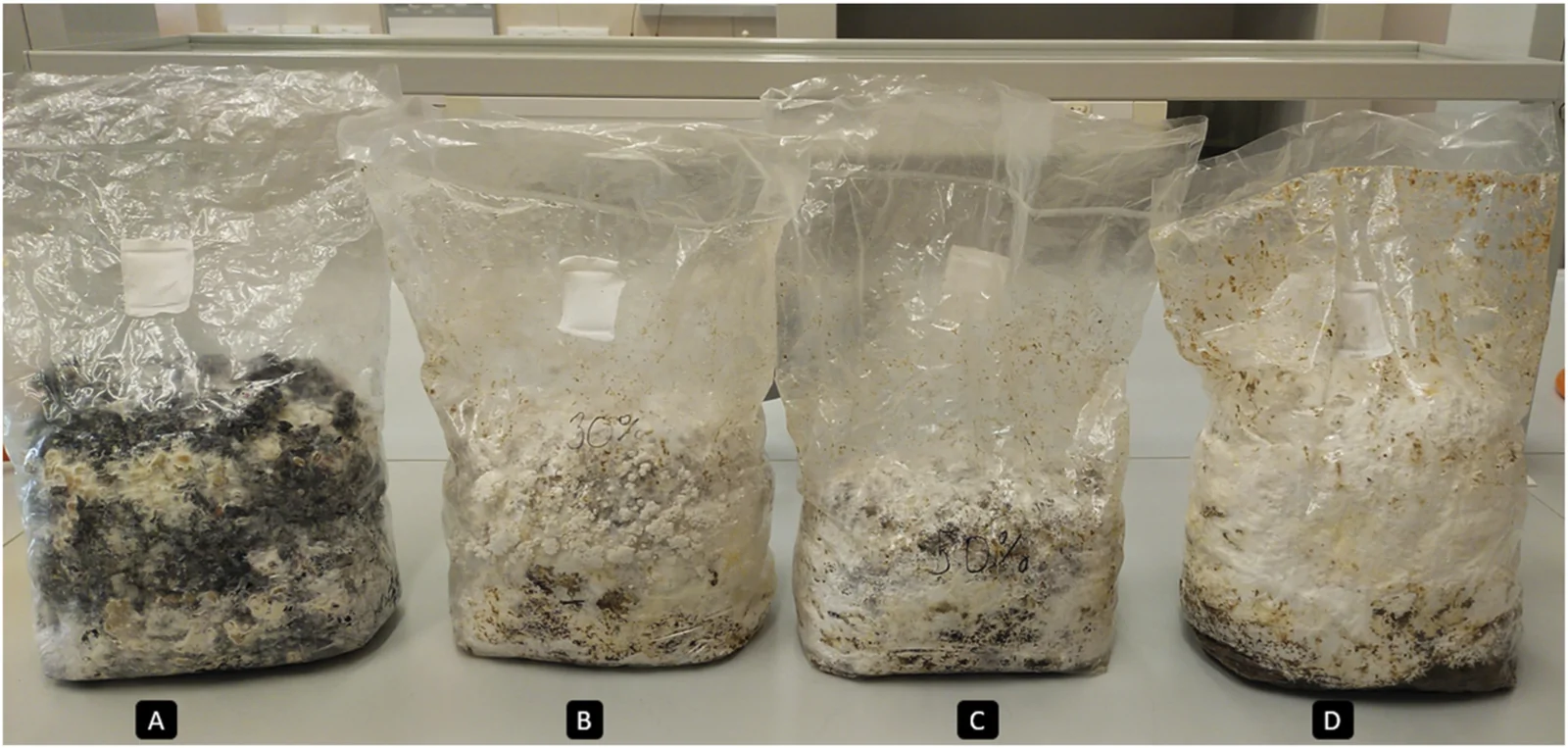
View of overgrown mycelium: **(A)** M100%TX; **(B)** M30%TX; **(C)** M50%TX; **(D)** M0%TX.

**FIGURE 4:**
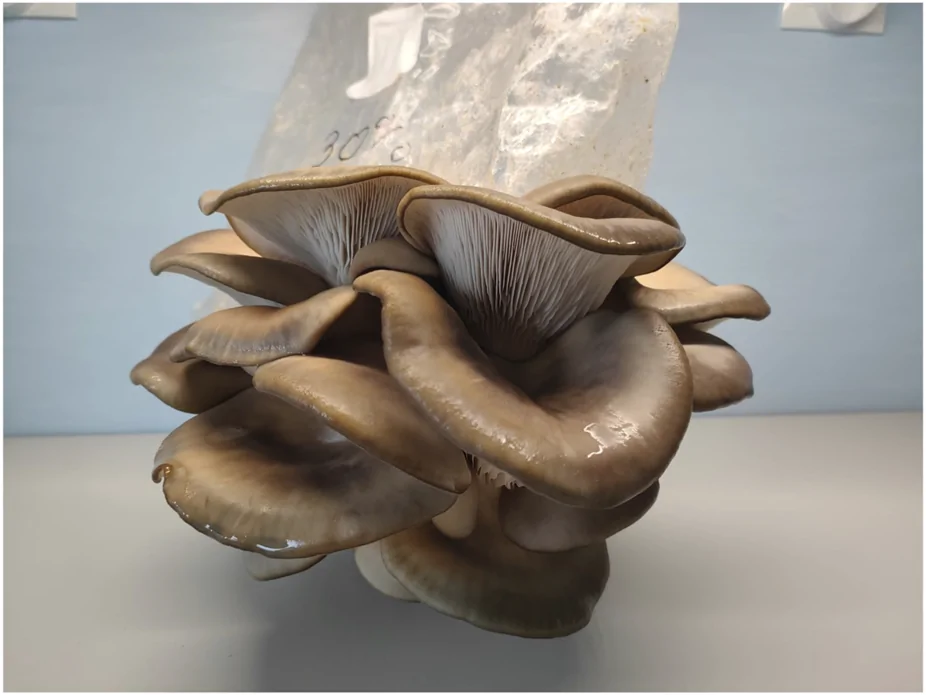
Oyster mushroom fruiting body grown on a textile substrate - M50%TX (50% oak wood pellets + 50% textile waste).

Images of the fruiting bodies differed significantly depending on the percentage of textile waste used in *P. ostreatus* cultivation (compare Figures 2A,F). Similarly, the structure and coloration of the mycelium varied according to the textile waste content in the substrate (Figures 2B–E). One may speculated that the darker coloration observed in samples with higher textile waste content resulted from chemical compounds used in textile dyeing.

Less compact mycelium was produced on a substrate containing 100% textile waste (Figure 3A). Based on the organoleptic assessment, the greater homogeneity of the contents in analytical bags B, C, and D indicates that the supplementation with oak pellets enhances uniform mycelial colonization of the substrate (Figures 3B–D).

The addition of textile waste to the substrate for *P. ostreatus* cultivation did not prevent the formation of fruiting bodies from the mycelium (Figure 4). However, the presence of textile waste reduced the yield of harvested fruiting bodies (Table 1).

SEM images of the fruiting bodies differed depending on the textile waste content (Figure 5). The most heterogeneous micrographs were observed in samples of fruiting bodies developed on substrate containing 50% textile waste (Figure 5, F50%). In contrast, fruiting body tissues grown without textile waste or with only 30% textile waste were relatively homogeneous (Figure 5, F0%, F30%, and F100%). It cannot be confirmed whether these structural features are representative of the entire fruiting bodies, as only small tissue fragments were analysed.

**FIGURE 5:**
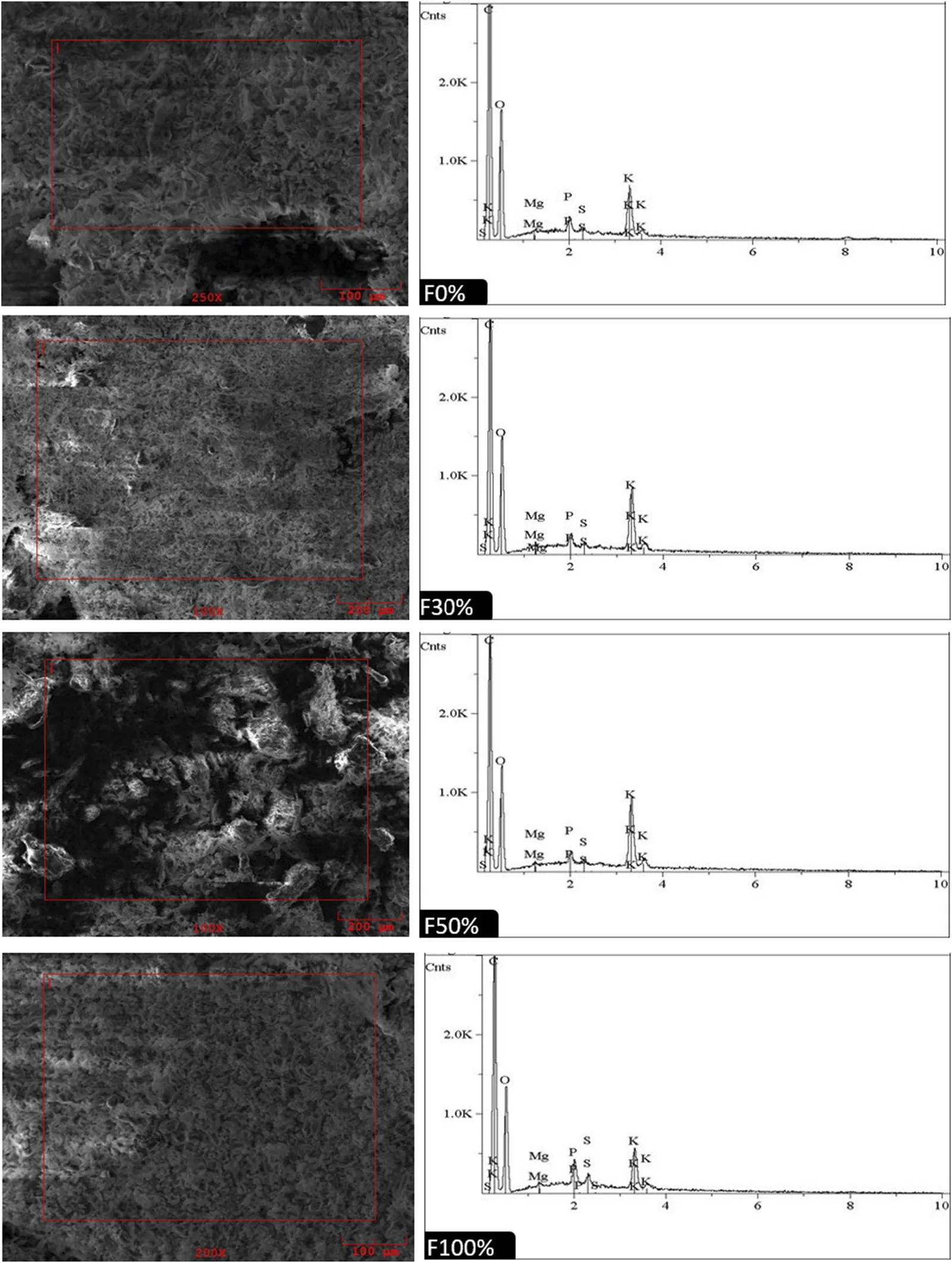
Sample SEM images and EDS spectra from selected areas of the tested samples. F0%, F30%, F50%, and F100%

Even greater variability in SEM images was observed in ash samples obtained after processing at 600 °C (Figure 6). The heterogeneity of ash micrographs increased with higher textile waste content (compare Figure 6, F30%–F100%).

**FIGURE 6:**
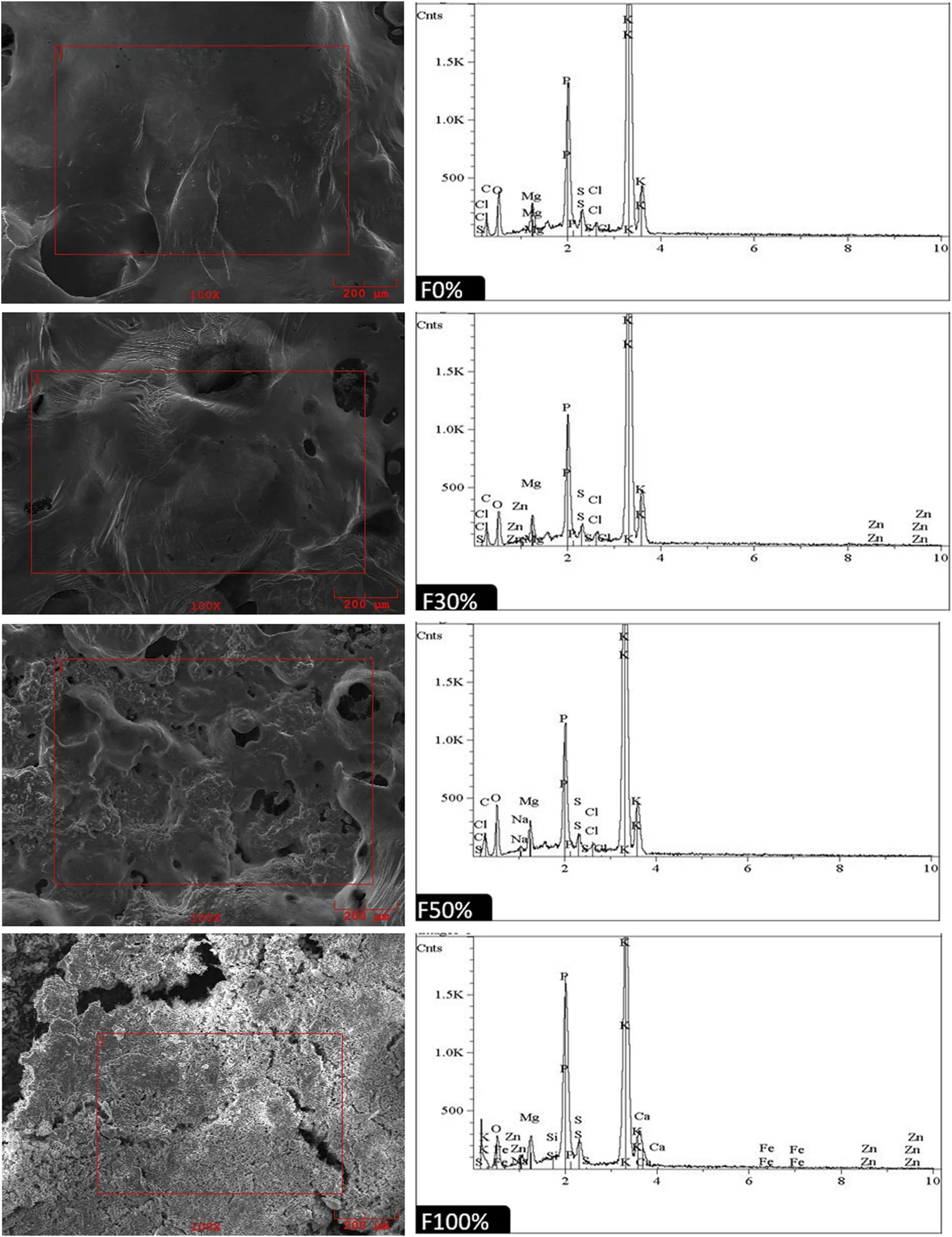
Sample SEM images and EDS spectra from selected areas of the tested ash samples. F0%, F30%, F50%, - ash 600 °C

Similarly, SEM micrographs of *P. ostreatus* mycelium varied depending on the textile waste content. The mycelium overgrew and decomposed the textile waste (see Figure 7M30%TX, M50%TX, M100%TX, and 100%TX). In contrast, a distinctly different image was observed for mycelium grown on pure oak substrate (Figure 7, M0M0%TX), where no fibrous textile structures were visible. SEM images of mycelial ash were much more homogeneous and did not change with textile waste content (Figure 8).

**FIGURE 7:**
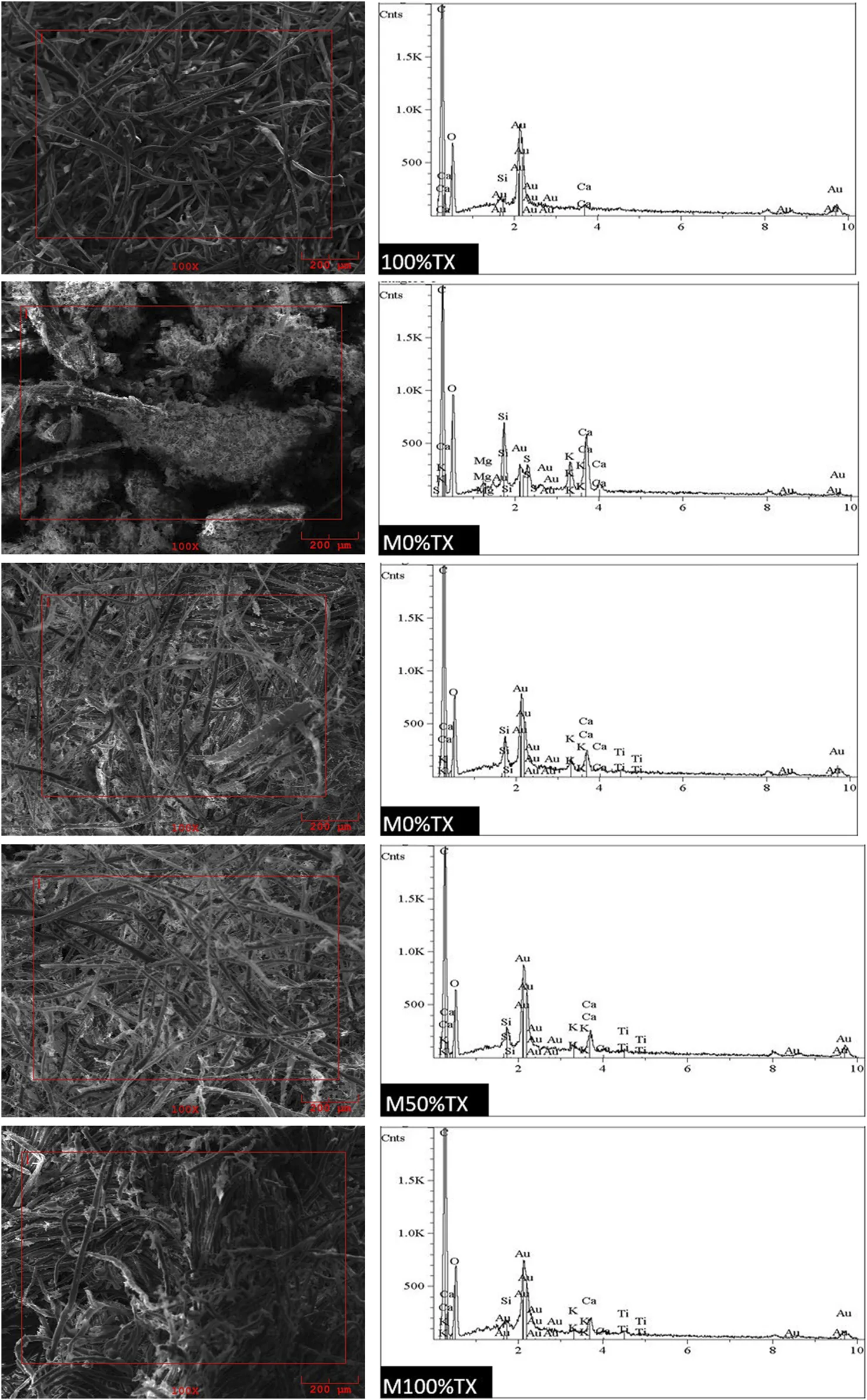
Sample SEM images and EDS spectra from marked areas of the tested samples 100%TX; M0%TX; M30%TX; M50%TX; M100%TX (after spraying the sample surface with gold).

**FIGURE 8:**
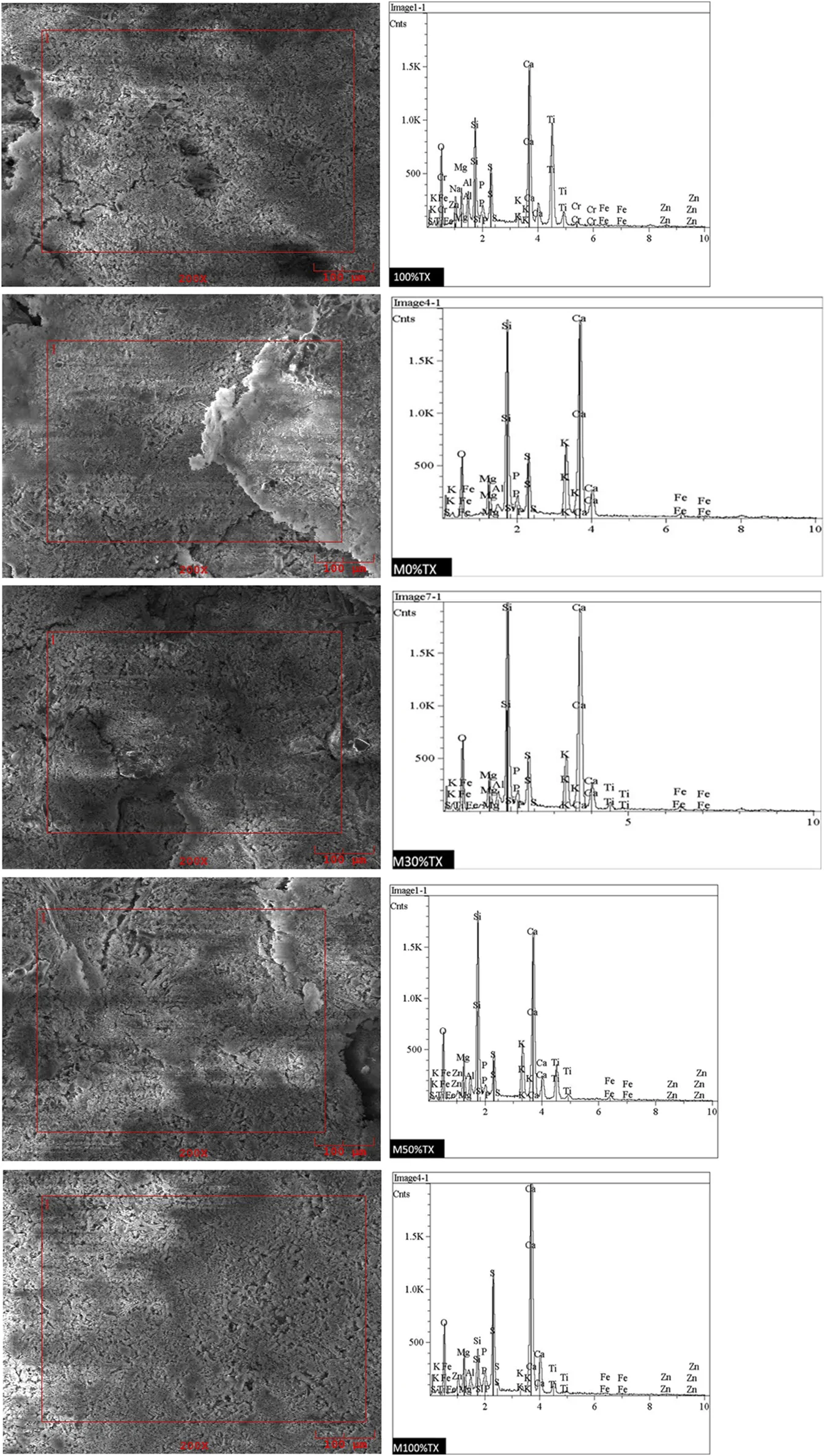
Sample SEM images and EDS spectra from marked areas of the tested ash samples 100%TX; M0%TX; M30%TX; M50%TX; M100%TX.

**FIGURE 9:**
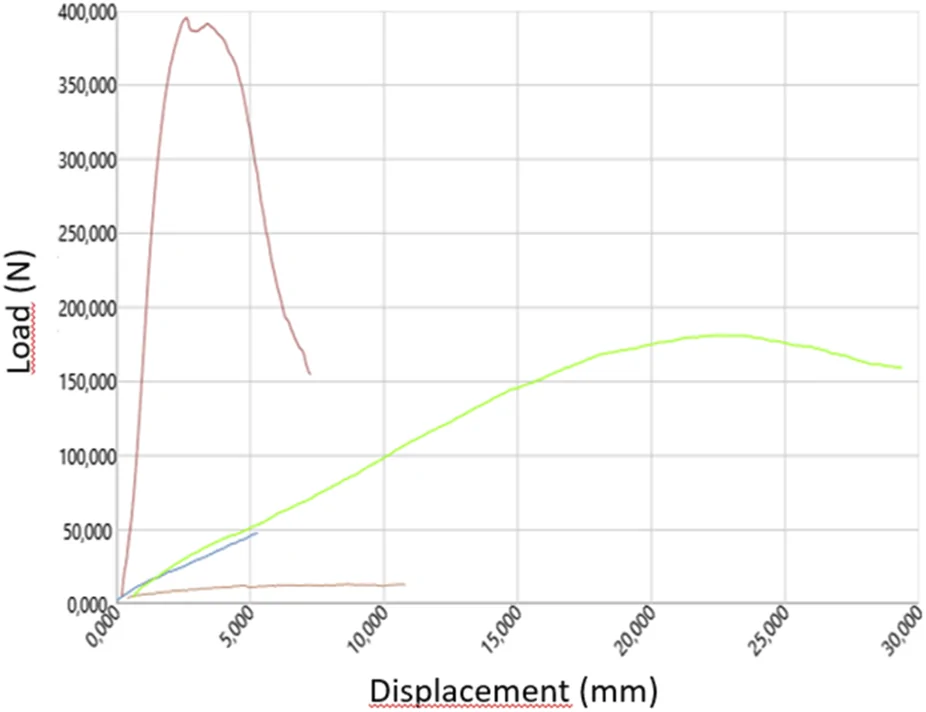
Comparison of displacement of textile-mycelium composites generated by *Pleurotus ostreatus* cultivation.

For each sample, three to four EDS spectra were collected from individual micro-areas of the surface. Qualitative analysis was based on the identification of characteristic X-ray lines corresponding to specific elements. The detection limit of the EDS method was approximately 0.3 wt% for elements with atomic numbers greater than sodium and approximately 0.5 wt% for lighter elements (from boron to sodium). Because measurements were performed locally on the sample surface, the results represent approximate elemental compositions dependent on the selected micro-areas. The gold (Au) peaks observed in the EDS spectra of the initial samples originated from the gold sputter coating applied prior to analysis.

#### 3.2.1 *Elemental composition of mycelium and textile* substrates

Semi-quantitative SEM/EDS results for ash samples obtained at 600 °C are presented in Table 2. Mycelium samples M0%TX, M30%TX, and M50%TX consisted predominantly of oxygen (O), calcium (Ca), silicon (Si), potassium (K), sulfur (S), and magnesium (Mg). In the M50%TX ash sample, titanium (Ti) was additionally detected. The M100%TX ash sample contained mainly O, Ca, Ti, Si, S, Mg, aluminum (Al), and sodium (Na). The ash derived from textile waste (100%TX) consisted primarily of O, Ca, S, Mg, Si, and Ti.

**TABLE 2:** Results of semi-quantitative analysis of element content in micro-areas of sample surfaces M0%TX; M30%TX; M50%TX; M100%TX; 100%TX - ash 600 °C.

SEM/EDS analysis [% by weight]					
Element	M0%TX ash 600 °C	M30%TX ash 600 °C	M50%TX ash 600 °C	M100%TX ash 600 °C	100%TX ash 600 °C
C	1.5	2.8	1.6	1.0	-
O	42.3	46.0	44.7	48.6	47.0
Mg	3.4	2.7	3.9	2.8	2.9
Al	0.7	0.9	1.3	0.8	2.5
Si	15.3	15.7	14.8	2.5	6.8
P	1.4	1.1	1.0	1.4	1.2
S	5.2	4.2	4.2	7.7	4.6
K	7.2	5.1	5.3	0.4	0.7
Ca	22.2	19.2	18.9	32.7	17.2
Ti	-	1.4	3.2	1.6	13.1
Fe	1.1	0.7	0.9	0.3	0.4
Zn	-	-	0.3	0.5	0.8
Na	-	-	-	-	2.2
Cr	-	-	-	-	0.6

Semi-quantitative SEM/EDS analysis of the initial (non-ashed) mycelium and textile samples is presented in Table 3. These samples were dominated by carbon (C) and oxygen (O), with minor contributions from calcium (Ca), potassium (K), silicon (Si), and magnesium (Mg).

**TABLE 3:** Results of semi-quantitative analysis of element content in micro-areas of sample surfaces M0%TX; M30%TX; M50%TX; M100%TX; 100%TX.

SEM/EDS analysis [% by weight]					
Element	M0%TX	M30%TX	M50%TX	M100%TX	100%TX
C	51.0	56.4	55.5	59.7	59.1
O	41.2	38.8	40.8	38.4	40.3
Mg	0.4	-	1.4	-	-
Si	2.7	1.8	-	0.3	0.3
S	0.4	-	-	-	-
K	1.5	0.1	0.6	0.1	-
Ca	2.9	1.9	1.6	1.1	0.4
Ti	-	0.1	0.2	0.3	-

#### 3.2.2 Elemental composition of fruiting bodies

SEM/EDS analysis of fruiting bodies (initial samples) revealed that carbon (C), oxygen (O), and potassium (K) were the predominant elements in all variants (F0%, F30%, F50%, and F100%), as summarized in Table 4. Minor amounts of magnesium (Mg), phosphorus (P), and sulfur (S) were also detected. Ash samples of fruiting bodies obtained after mineralization at 600 °C were analyzed by SEM/EDS, and the results are shown in Table 5. The ash was composed mainly of potassium (K), oxygen (O), phosphorus (P), carbon (C), and magnesium (Mg). In the F100%TX ash sample, additional elements such as sulfur (S), calcium (Ca), zinc (Zn), iron (Fe), and silicon (Si) were detected.

**TABLE 4:** Results of semi-quantitative analysis of element content in micro-areas of sample surfaces F0%, F30%, F50%, and F100%.

SEM/EDS analysis [% by weight]				
Element	F0%TX	F30%TX	F50%TX	F100%TX
C	52.5	52.3	51.9	55.3
O	44.0	43.7	43.6	40.4
Mg	0.1	0.2	0.1	0.2
P	0.6	0.5	0.5	1.1
S	0.2	0.1	0.2	0.5
K	2.5	3.2	3.7	2.6

**TABLE 5:** Results of semi-quantitative analysis of element content in micro-areas of sample surfaces F0%, F30%, F50%, and F100% - ash (600 °C).

SEM/EDS analysis [% by weight] ash				
Element	F0%TX	F30%TX	F50%TX	F100%TX
C	5.9	7.6	9.4	​
O	37.1	34.5	35.7	35.1
Mg	2.7	2.7	2.3	3.1
P	11.0	9.7	9.0	18.6
S	1.4	1.1	1.1	2.3
Cl	0.5	0.7	0.5	​
K	41.4	43.0	​	36.2
Na	​	0.5	0.4	1.3
Zn	​	0.4	​	0.5
Si	​	​	​	0.3
Ca	​	​	​	2.3
Fe	​	​	​	0.2

#### 3.2.3 Ash content determination

The results of ash content determination by gravimetric analysis are presented in Table 6. All analyzed samples were predominantly organic, with inorganic (ash) content ranging from approximately 1%–13% by mass. The highest ash content was observed for mycelium grown on 100% oak wood pellets (M0%TX), while the lowest ash content was measured for the textile waste sample (100%TX).

**TABLE 6:** Results of ash content determination.

Sample	Content, % (m/m)	
	Ash (600 °C)	
M0%TX	13.10	13.0^*^ ^)^
	12.80	
M30%TX	9.00	8.6^*^ ^)^
	8.10	
M50%TX	6.30	. 6.1^*^ ^)^
	5.90	
M100%TX	6.40	6.2^*^ ^)^
	6.00	
100% TX	1.25	1.1^*^ ^)^
	1.00	
I0%	5.1	5.1^*^ ^)^
	5	
I30%	5.7	5.7^*^ ^)^
	5.7	
I50%	6.1	6.1^*^ ^)^
	6.1	
I100%	5.3	5.3^*^ ^)^
	5.3	

* ) arithmetic mean of the results obtained from the analysis of two parallel analytical samples +/-0.1

The samples exhibited heterogeneity, as indicated by differences between parallel determinations that exceeded the uncertainty of the gravimetric method. Therefore, the average values reported in Table 6 should be regarded as approximate estimates of ash content.

#### 3.2.4 FAAS analysis of metal content

Quantitative determination of selected metals was performed using FAAS following microwave digestion. The results for the fruiting bodies grown on substrates containing textile waste are summarized in Table 7. Zinc (Zn), iron (Fe), and copper (Cu) were detected in all fruiting body samples. Their concentrations increased with increasing textile waste content, reaching maximum values of 170 mg/kg Zn, 80 mg/kg Fe, and 21 mg/kg Cu in the fruiting bodies cultivated on 100% textile waste (F100%). In contrast, cadmium (Cd), cobalt (Co), nickel (Ni), and lead (Pb) remained below the quantification limits of the FAAS method in all fruiting body samples. Manganese (Mn) was also below the quantification limit (<20 mg/kg) in the fruiting bodies, with only negligible signals detected occasionally at the baseline.

**TABLE 7:** Results of quantitative analysis (FAAS method) for metal content in the fruiting bodies.

Metal	F0% [mg/Kg]	F30% [mg/Kg]	F50% [mg/Kg]	F100% [mg/Kg]
Zinc (Zn)	48*)	50*)	71*)	170*)
Iron (Fe)	39*)	32*)	52*)	80*)
Copper (Cu)	10*)	8*)	11*)	21*)
Cadmium (Cd)	<5*)**)	<5*)**)	<5*)**)	<5*)**)
Cobalt (Co)	<20*)**)	<20*)**)	<20*)**)	<20*)**)
Manganese (Mn)	<20*)**)***)	<20*)**)***)	<20*)**)***)	<20*)**)***)
Nickel (Ni)	<20*)**)	<20*)**)	<20*)**)	<20*)**)
Lead (Pb)	<20*)**)	<20*)**)	<20*)**)	<20*)**)

*) arithmetic mean of the results obtained from the analysis of two parallel analytical samples (sample weights), **) limit of quantification of the test method used

***) during the measurement, slight signals were detected at the detection limit of the method

The quantitative distribution of metals within the isolated mycelium and raw textile waste is presented in Table 8. Zinc, iron, copper, and manganese were successfully quantified in the mycelium samples. Notably, the mycelium exhibited a distinct capacity for iron bioaccumulation, showing concentrations ranging from 1000 mg/kg in the wood pellet control (M0%TX) down to 190 mg/kg in the pure textile variant (M100%TX).

**TABLE 8:** Results of quantitative analysis (FAAS method) for metal content in the myceliu.

Metal	M0%TX [mg/Kg]	M30%TX [mg/Kg]	M50%TX [mg/Kg]	M100%TX [mg/Kg]	100% TX [mg/Kg]
Zinc (Zn)	62*)	71*)	120*)	390*)	130*)
Iron (Fe)	1000*)	590*)	440*)	190*)	48*)
Copper (Cu)	<10*)**)***)	11*)	25*)	50*)	25*)
Cadmium (Cd)	<5*)**)	<5*)**)	<5*)**)	<5*)**)	<5*)**)
Cobalt (Co)	<20*)**)	<20*)**)	<20*)**)	<20*)**)	<20*)**)
Manganese (Mn)	110*)	70*)	53*)	32*)	<20*)**)***)
Nickel (Ni)	<20*)**)***)	<20*)**)***)	<20*)**)***)	<20*)**)***)	<20*)**)
Lead (Pb)	<20*)**)	<20*)**)	<20*)**)	<20*)**)	<20*)**)

* ) arithmetic mean of the results obtained from the analysis of two parallel analytical samples (sample weights),**) limit of quantification of the test method used, ***) during the measurement, slight ignals were detected at the detection limit of the method.

It is important to address the apparent operational divergence between the bulk FAAS quantification and the semi-quantitative SEM/EDS scanning. While Fe was not registered in the raw, non-ashed mycelium spectra (Table 3), this is directly attributable to the detection constraints of the EDS microanalyzer. The highest Fe value of 1000 mg/kg corresponds to a mere 0.1 wt%, which falls at the absolute lower detection threshold of EDS when masked by a massive organic carbon/oxygen matrix (>95 wt%). Once the organic mass was eliminated via thermal mineralization at 600 °C, the inorganic ash components were concentrated approximately ten-fold (consistent with the gravimetric ash data in Table 6). This concentration effect allowed the accumulated iron to be readily quantified in the ash samples at 0.3–1.1 wt% (Table 2), validating the structural insights provided by both analytical methodologies. Furthermore, unlike the fruiting bodies, the mycelium actively retained quantifiable levels of manganese, which decreased from 110 mg/kg (M0%TX) to 32 mg/kg (M100%TX) as the proportion of textile waste expanded.

### 3.3 Mechanical properties of textile-mycelium composites

#### 3.3.1 Mechanical properties and testing limitations of textile-mycelium composites

The mechanical performance data under structural loading configuration are compiled in Table 9. It is critical to contextualize these findings within the structural boundaries and sensitivity limitations of the exploratory testing setup. The specimens were evaluated as thick, bulk sections (450–460 mm in length and 21 mm in thickness). Due to the highly compliant, porous, and foam-like nature of these mycelium-textile matrices, the initial yield responses fell near the baseline sensitivity threshold of the high-capacity load cell utilized. This experimental limitation explains the highly compressed stress readings ( $0.0\text{ to }0.1{\;N/\text{mm}}^{2}$ ) for the Styrofoam control and the 100% wood samples, as well as the unquantifiable modulus of elasticity ( E ) for several textile-dense replicates (denoted as n.d. in Table 9).

**TABLE 9:** Mechanical properties of textile-mycelium of *Pleurotus ostreatus* composites.

Substrate composition	Mean (x̄)	ME (95%)	Confidence interval (95%)
Percentage strain at the yield point — εm [%]			
Styro	5.60	0.24	[0.05; 0.06]
100%	3.40	0.15	[0.03; 0.04]
50%	2.50	0.11	[0.02; 0.03]
30%	n.d	n.d	n.d
Modulus of elasticity — E [N/mm2]			
Styro	0.40	0.02	[0.00; 0.00]
100%	6.20	0.27	[0.06; 0.06]
50%	0.10	0.00	[0.00; 0.00]
30%	0.50	0.02	[0.00; 0.01]
Maximum load — Fm rzeczywiste [N]			
Styro	49.40	2.14	[0.47; 0.52]
100%	396.60	17.23	[3.79; 4.14]
50%	14.90	0.65	[0.14; 0.16]
30%	183.10	7.96	[1.75; 1.91]
Load at maximum deformation — F(Def1Max) [N]			
Styro	48.30	2.10	[0.46; 0.50]
100%	154.30	6.71	[1.48; 1.61]
50%	12.20	0.53	[0.12; 0.13]
30%	159.10	6.91	[1.52; 1.66]

* n.d. = not determined due to load-cell sensitivity thresholds and localized matrix compaction during thick-specimen deformation.

**TABLE 10:** Analysis of thermal parameters of composites.

Mycelium	T_5_ (°C)	T_RMA X1_ (°C)	T_RMA X2_ (°C)	dm/dt 1 (%/min)	dm/dt 2 (%/min)	ΔT_s_ (°C)	P_600_ (%)
M100%TX	270	330	430	2.6	14.3	390–600	2
M50%TX	230	300	450	7.5	4.6	420–600	7
M30%TX	245	320	410	4.1	6.5	375–600	9
M0%TX (Ref.)	215	286	460	6.1	3.7	420–600	15

Furthermore, while the structural behavior displays elastic deformation and high displacement reminiscent of flexible foam insulation, standard plastic present clear geometric limitations for thick bio-composites. The dense, interwoven network of post-consumer textile fibers provides an accumulation of micro-slip planes rather than a homogeneous crystalline stress distribution. Consequently, the data points missing from Table 9 represent regions where the displacement occurred via progressive localized compaction rather than a single measurable elastic yield point. Future characterizations must utilize specialized low-capacity load cells and foam-specific testing frameworks (e.g., ISO 844 or ASTM D1621 for compressive properties) on optimized sample geometries to fully isolate the individual elastic components of the hybrid matrix.

### 3.4 Thermal analysis of mycelium-textile composites

Based on the results of TG and DTG curve analysis of the tested composites made of mycelium in an oxygen atmosphere (Figure 10), it was found that all samples undergo two-stage thermal decomposition. The first stage of thermal decomposition occurs in the temperature range of 280 °C–340 °C, while the second, main stage of decomposition is observed at higher temperatures and covers the range of 410 °C–460 °C, depending on the composition of the composite. The second stage of decomposition, which is dominant in terms of mass loss, is characterized by distinct minima on the DTG curves for M100%TX and M30%TX. For the reference sample, the maximum degradation rate in the second stage is observed at a temperature of approximately 460 °C, while for mycelium-modified composites, the TRMAX2 values shift to lower temperatures, with a simultaneous change in the dm/dt parameter value. The decrease in the rate of mass loss, expressed by a decrease in the absolute value of the dm/dt parameter, observed with an increase in the content of carbon filler in the polymer matrix, indicates a reduction in the amount of volatile and combustible decomposition products released from the sample. The highest intensity of the second stage of decomposition, expressed by the value of dm/dt2, was recorded for M100%TX.

**FIGURE 10:**
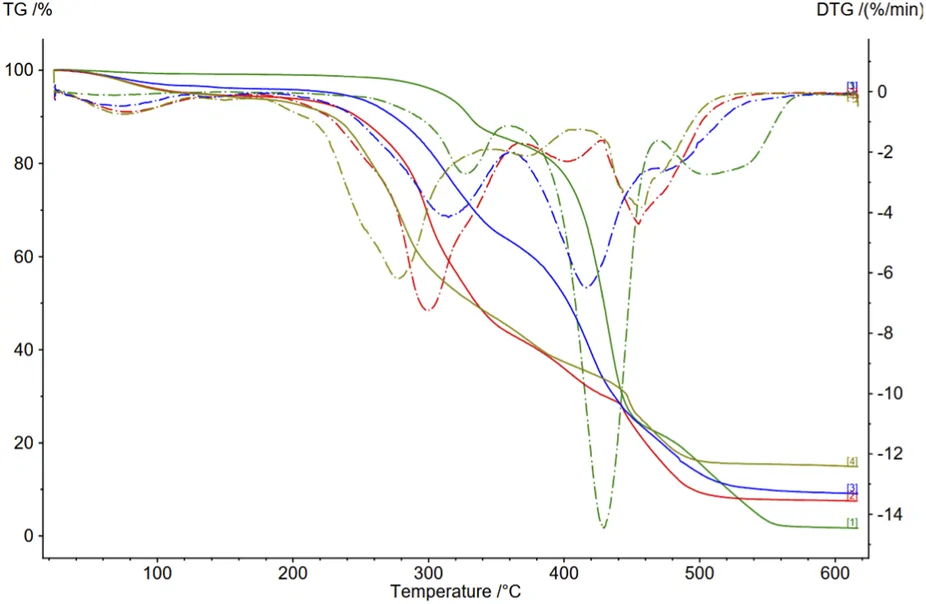
TG and DTG analysis of fungal composites: 1- M100%TX, 2 - M50%TX, 3 - M30%TX, 4- M0%TX.

Analysis of the results obtained indicates that the addition of mycelium significantly affects the thermal stability of the tested textile composites. Modification of waste with mycelium leads to a shift in the initial decomposition temperature (T_5_) towards higher values compared to the reference sample, for which T5= 215 °C, while for M30%TX, M50%TX, and 1 M100%TX 00% mycelium, the T5 index increases to 245, 230, and 270 °C, respectively. This phenomenon indicates a reduction in the rate of thermal decomposition in the initial stage of the process. The total mass loss (PTD) of the tested materials ranges from 6 to 19% and decreases with increasing textile waste content. At the same time, a significant reduction in mass residue at 600 °C (P600) is observed from 15% for the reference sample to 2% for the composite containing 100% textiles. This change indicates that the incorporation of textile waste affected the initial stage of thermo-oxidative degradation of the composites. The reduced residue after thermal decomposition is most likely associated with the different chemical composition of the textile-based substrates, including the presence of polyester, cotton, nylon, elastane, viscose/lyocell, and textile additives. These components differ from oak wood pellets in terms of their thermal decomposition pathways and mechanisms. Consequently, the use of a heterogeneous textile matrix influences both the onset of thermal degradation and the values of the other parameters determined from TG/DTG analysis. The metal content of the substrates was not directly determined prior to inoculation; therefore, the relationships concerning the bioavailability of Fe, Zn and Cu should be regarded as a hypothesis requiring confirmation in future studies. Further work should include C:N analysis, determination of elements in the starting substrates, and studies on the leachability of metals from the composites.

## 4 Discussion

Results of the present study demonstrate that mixed textile waste can be effectively utilized as a component of cultivation substrates for *Pleurotus ostreatus*, supporting both mycelial growth and fruiting body formation. Substrate formulations containing 30%–50% textile waste combined with oak wood pellets were particularly suitable, providing a balance between biological efficiency and composite formation. Although substrates composed entirely of textile waste supported fungal growth, they exhibited reduced fruiting body yields compared to wood-based controls (Cerimi et al., 2019; Pathak and Navneet, 2017; Harmsen et al., 2021).

The observed decrease in biological efficiency with increasing textile content is consistent with previous reports indicating that lignocellulosic substrates rich in cellulose and hemicellulose are more favourable for mushroom fruiting than synthetic polymer-rich materials. Oak wood pellets likely provided essential nutrients and structural properties that enhanced mycelial colonization and fruiting body development. In contrast, higher proportions of synthetic fibres may have limited nutrient availability or altered substrate porosity, resulting in reduced yields (Zhao et al., 2021).

Metal analysis revealed that zinc and iron were the dominant metals detected in both mycelium and fruiting bodies, while copper was present at trace levels (Figure 11).

**FIGURE 11:**
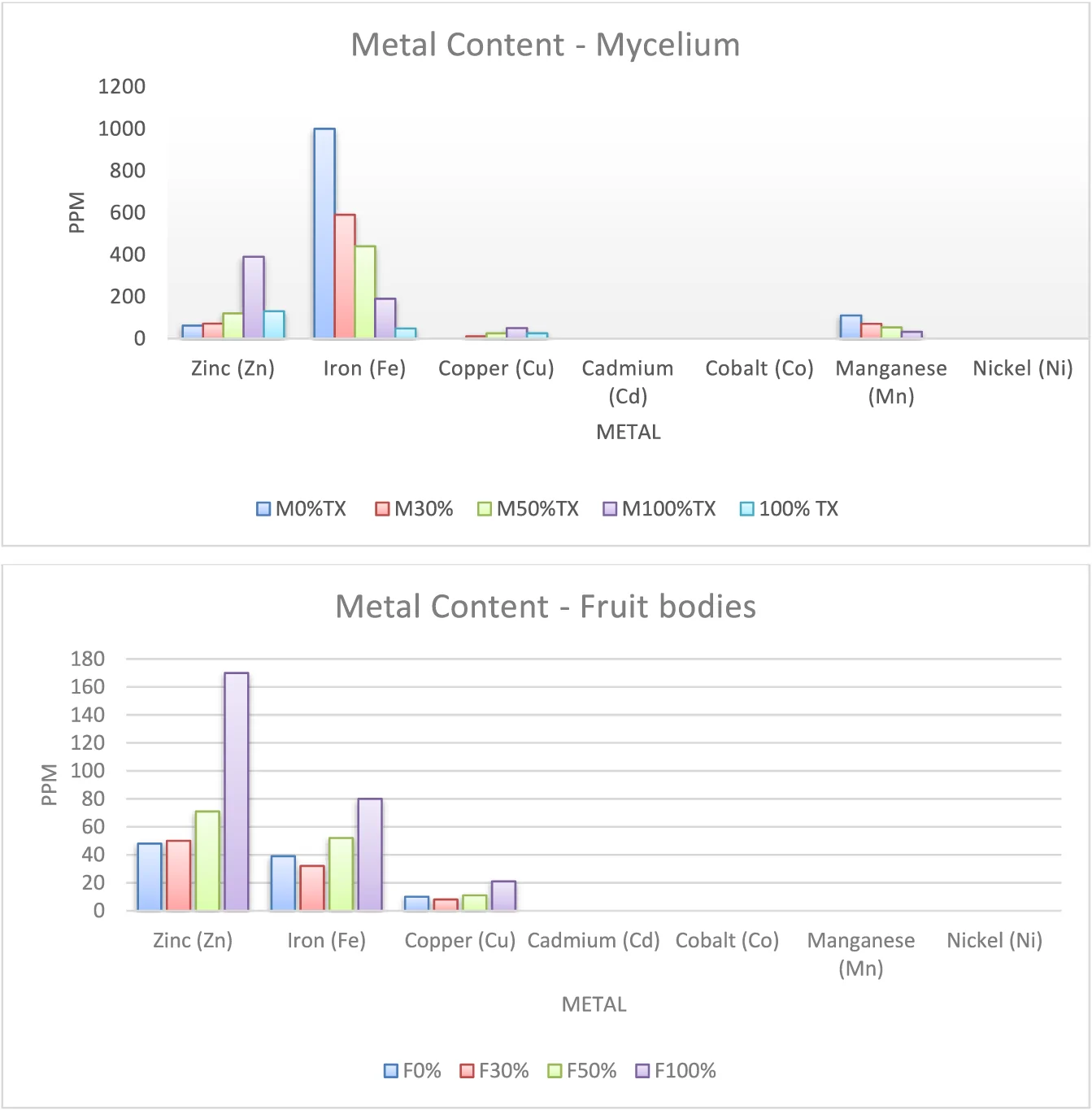
Metal content in mycelium and fruit bodies of *Pleurotus ostreatus* cultivated on textile waste and oak pellets.

Importantly, the concentrations of essential trace elements such as Zn, Fe, and Cu in the fruiting bodies and mycelium reflect the baseline mineral profile of the cultivated biomass, rather than a toxicological hazard. While toxic heavy metals (such as cadmium, lead, cobalt, and nickel) remained below the detection limits of the FAAS method, the elemental composition serves primarily to characterize the macro- and micronutrient distribution within the structural matrix. Consequently, these mineral levels—alongside the major ash components like Potassium, Phosphorus, and Magnesium—should be evaluated strictly in terms of their influence on fungal metabolism, hyphal binding efficiency, and the overall biological properties of the resulting textile-mycelium biomaterials, rather than for dietary safety.

SEM/EDS analysis indicated that calcium was a major inorganic component of the mycelium, whereas it was largely absent from the fruiting bodies. In contrast, FAAS analysis identified zinc and iron as the predominant metals accumulated in fruiting bodies. These observations suggest differential metal uptake and translocation mechanisms between mycelium and fruiting bodies. Such selective bioaccumulation has been reported previously for *Pleurotus* species (Kapahi and Sachdeva, 2017) and may be influenced by physiological metal transport systems within the fungal organism.

The reduction in biological efficiency with increasing textile content was strongly non-linear. Replacing oak wood pellets with 30% textile waste lowered BE from 55.18% (control) to 28.50% whereas further increases to 50% and 100% textile produced only modest additional declines (to 23.84% and 19.33%, respectively). The same trend was apparent between successive flushes: the second flush of the wood-based control retained about 93% of the first-flush yield, but this proportion fell progressively with textile content, to roughly 58%, 54%, and 48% for the 30%, 50%, and 100% textile substrates, respectively. Textile-rich substrates therefore not only produced lower yields per flush but were also less able to sustain repeated fruiting.

Several factors may account for this pattern. Oyster mushroom productivity is closely related to the amount and accessibility of degradable lignocellulose and by the C/N ratio and physical structure of the substrate (Sözbir et al., 2015; Zhao et al., 2021). The synthetic fibres that dominate the textile fraction (polyester and nylon) are essentially hard or not degraded by *P. ostreatus* during the cultivation cycle and supply neither carbon nor nitrogen. They act as an inert filler that dilutes the nutritionally available matrix and alters porosity, water retention, and aeration. Because the cotton fraction (∼25%) is cellulosic and thus. Additional textile mainly replaces already poor material, which would explain the plateau observed at higher textile contents. Dyes, finishing agents, and inorganic additives present in post-consumer textiles including the titanium detected by SEM/EDS, most probably as TiO_2_ may additionally exert mild inhibitory effects on mycelial metabolism. The present data do not allow these mechanisms to be disentangled, and dedicated experiments (e.g., comparing scoured textiles with untreated material) would be needed to quantify their relative contributions.

The relationship between metal content and textile waste proportion was complex. Increasing textile content generally resulted in higher zinc concentrations in both mycelium and fruiting bodies, particularly in the 100% textile substrate. Conversely, iron concentrations in fruiting bodies decreased with increasing textile content, suggesting that oak wood pellets may be a more significant source of iron than textile waste. This inverse relationship highlights the importance of substrate composition in determining metal bioavailability and uptake.

The presence of textile dyes and finishing chemicals may also influence metal accumulation patterns. Although not directly investigated in this study, dyes and auxiliary chemicals present in post-consumer textiles could affect metal binding, solubility, and transport within the fungal biomass. This aspect warrants further investigation to better understand the interactions between textile chemistry and fungal metabolism (Kohphaisansombat et al., 2024; Elsacker, Vandelook, and Peeters, 2023) It should also be noted that standard autoclaving (121 °C) is unlikely to remove such compounds and may, in some cases, mobilize or transform them (e.g., formaldehyde release from crease-resistant resin finishes). Their fate during substrate sterilization, and any effects on mycelial growth or product safety, were not examined here and warrant dedicated investigation (Rovira et al., 2025).

Mechanical testing of textile–mycelium composites demonstrated that their properties are strongly dependent on substrate formulation. Composites containing 30% textile waste exhibited favourable elastic deformation behaviour and mechanical performance comparable to or exceeding that of conventional Styrofoam insulation materials. Increasing the textile content to 50% resulted in a marked reduction in displacement and mechanical resilience, while composites derived solely from oak wood pellets showed high resistance to applied stress but limited deformation capacity (Bitting et al., 2022; Stelzer et al., 2021)These findings indicate that the mechanical and thermal properties of textile–mycelium composites can be tailored by adjusting the ratio of textile waste to lignocellulosic material (Freeman et al., 2024)This tunability offers significant potential for the development of bio-based construction and insulation materials with customized performance characteristics (Sangosanya and Pistofidou, 2024; Zhang et al., 2024; Tian et al., 2022).

The introduction of post-consumer textile waste into the oak pellet substrate not only alters the biological parameters of the cultivation but also plays a critical role in the potential structural applications of the resulting biocomposites. Literature indicates that the hybridization of textile fibers with lignocellulosic agricultural residues can serve as an effective reinforcement strategy for the mycelial matrix. As demonstrated by Ruiz et al. (2024) in their study on composites utilizing *P. ostreatus* and *G. lucidum*, a systematic approach to mechanical evaluation using standardized methods (e.g., ASTM C165, D1621) reveals compressive strengths reaching up to 775 kPa under 30% deformation. While the mechanical evaluation in the present study was qualitative, implementing standardized strength testing frameworks, alongside alternative post-processing methods—such as the non-thermal saline solution deactivation proposed by Ruiz et al. (2024)—represents a logical and planned direction for future work to optimize these textile-mycelium materials.

Overall, the present study aligns with previous research demonstrating the feasibility of using fungal mycelium as a sustainable material platform. Compared with earlier studies focusing on agricultural residues or pure plant fibres, the incorporation of mixed textile waste represents an innovative approach to addressing the growing environmental burden associated with post-consumer textiles.

A key characteristic of a genuine circular economy strategy is the comprehensive evaluation of material degradability and its subsequent environmental impact at the end-of-life stage. While the organic fraction of the textile-mycelium composite—composed of fungal chitin, wood lignin, and natural cotton cellulose—is inherently biodegradable under typical microbial action, the ultimate fate of the bioaccumulated contaminants presents a significant environmental bottleneck. The inorganic elements retained by the biomass, such as the titanium complexes (reaching 3.2 wt% in the M100%TX ash) and persistent synthetic organic textile pigments, do not biologically degrade.

During open-environment composting or landfilling, the natural decomposition of the organic matrix would inevitably result in the secondary leaching and release of these non-biodegradable additives into the soil and groundwater systems. Consequently, to prevent eco-toxicity hazards, these specific composites cannot be categorized as freely compostable materials. Instead, their circular lifecycle must be restricted to managed, closed-loop technical cycles aiming to maximize the product’s lifespan and minimize waste generation. The mechanical and thermal results indicate that textile–mycelium composites have characteristics that justify further investigation as bio-based insulation materials. However, the present experiments should be considered preliminary. Direct comparison with conventional insulation products such as expanded polystyrene requires standardized testing of density, thermal conductivity, compressive properties, dimensional stability, moisture resistance, fire behaviour and long-term durability. Therefore, the present study demonstrates material potential rather than equivalence to commercially certified insulation materials.

## 5 Conclusion

This study demonstrates that mixed post-consumer textile waste can be effectively valorized through *Pleurotus ostreatus* cultivation, enabling both mushroom production and the formation of mycelium–textile biocomposites. Substrates containing 30%–50% textile waste combined with oak wood pellets supported robust mycelial growth and satisfactory fruiting body yields, while 100% textile substrates, although viable, resulted in reduced biological efficiency.

Elemental analysis confirmed that zinc, iron, and copper were the primary metallic elements accumulated within the mycelium and fruiting bodies. Rather than indicating suitability for dietary consumption, these quantified concentrations characterize the baseline mineral profile embedded within the structural biomass. These essential trace elements, together with the primary macronutrients detected in the ash matrix, play a functional role in defining the biological properties and metabolic activity of the fungus during the colonization of the textile-containing substrates.

The differential distribution of metals between mycelium and fruiting bodies indicates selective uptake and translocation mechanisms influenced by substrate composition.

Mechanical testing showed that textile–mycelium composites exhibit tuneable properties depending on textile content. However, the current mechanical and thermal measurements should be regarded as preliminary characterisation rather than validation in comparison with conventional insulation products. Further work should include standardised mechanical and thermal tests, characterisation of density and moisture content, and an assessment of fire resistance. These findings highlight the potential of fungal biocomposites as sustainable alternatives for building applications - a promising circular-economy strategy. Further research into the mycelium-textile composite will include an expanded scope of analysis covering textile waste from the pre-consumer stage; the impact of dyes and finishing agents used in textile materials on the rate and pattern of mycelium growth will be analysed; and the demand for elements will be determined.

## Data Availability

The raw data supporting the conclusions of this article will be made available by the authors, without undue reservation.
